# “You’re Never Really Safe”: Exploring UK Mothers’ and Young Women's Perceptions of Sexual Violence Risk

**DOI:** 10.1177/10778012241289434

**Published:** 2024-11-04

**Authors:** Becca Wood, Sheri Fabian

**Affiliations:** School of Criminology, 1763 Simon Fraser University, Burnaby, BC, Canada

**Keywords:** sexual violence, altruistic fear, maternal perceptions, victimization, fear of crime

## Abstract

Studies exploring perception-based sexual violence risk across the United Kingdom are limited, but research that examines how mothers consider their daughter's risk is even more uncommon. This interview-based study compared the experiences of 10 mothers with 10 young women based in the United Kingdom to understand their perceived sexual violence risk, and how they manage such risks. Findings indicate conflicting ideas of risk perception, yet similarities across risk management strategies, and concerns over the UK government's ability to respond to sexual violence. Prevalence of sexual violence fear, current flaws within policy, and the importance of further studying the mother–daughter dyad are highlighted.

Rates of sexual violence across the United Kingdom have been increasing; by the end of 2023, police-recorded victimization had surpassed prepandemic levels—demonstrating a 17% increase in the number of police-recorded incidents (Jones, 2022). Despite the prevalence of sexual violence and fear of victimization (Crosby et al., 2022; Mellgren & Ivert, 2018; Silva & Wright, 2014), little is known about the impact of fear across women in the United Kingdom (UK). Furthermore, the government has made minimal efforts to address these increasing rates of sexual violence (Anitha & Lewis, 2018; Murray, 2021; Stöckl & Quigg, 2021). Studies of self-implemented risk management strategies among women have identified avoidance and self-precautionary measures, such as only traveling in groups, avoiding walking to certain locations, and making more conscious clothing choices (Ceccato & Loukaitou-Sideris, 2021; Gordon & Riger, 1989; Jackson & Gray, 2010; Stark & Meschik, 2018). Despite these findings, a significant gap in understanding other risk management strategies such as improvised weapons and the use of phones remains. Furthermore, no current research explores how this fear exists between women within families, specifically across the mother–daughter relationship. Research shows that older women are more likely to fear other crime types, such as burglary, over sexual violence (Mellgren & Ivert, 2018). However, literature has not addressed whether older women's fear of crime, specifically sexual violence victimization, transfers to fears for their daughters. The current study fills these gaps by examining and comparing sexual violence fear across mothers and young women in the UK, while also identifying how these groups mitigate their fear by proposing and adopting risk management strategies.

## Literature Review

### Prevalence of Sexual Violence

Sexual violence encapsulates gender-based violence, sexual harassment, and rape among many other behaviors (Graaf, 2021; Lewis et al., 2021). Across the UK, sexual violence rates have been rising (Jones, 2022); statistics show that one in four women reported being raped or sexually assaulted as an adult, and by the end of December 2021, police recorded the highest number of rapes to date at 67,125 (Rape Crisis England & Wales, 2022). However, the true prevalence of this violence is undetermined due to disparities across police-reported crimes, general social survey data, and underreporting of sexually violent incidents (Persson & Dhingra, 2020; Silva & Wright, 2014).

Crime statistics and empirical evidence show that women are more likely to be victimized by an acquaintance rather than a stranger (Persson & Dhingra, 2020). Despite this, national data identified that 15% of police-reported sexually violent crimes were perpetrated by a stranger (Stripe, 2021), and one in 10 women were victimized in a public space (Stripe, 2021). Despite statistical evidence, women routinely report fearing stranger rape at a significantly higher level than sexual violence perpetrated by someone known to them (Boyer, 2022; Hickman & Muehlenhard, 1997).

### Fear, Risk, and Management

Women's fear of sexual violence is widespread and often leads to increased anxiety in their daily lives (Crosby et al., 2022; Mellgren & Ivert, 2018; Silva & Wright, 2014). Women experience significantly higher amounts of fear relating to their own victimization compared to men (Cisneros, 2019; Jackson, 2009; Lane, 2012; Mellgren & Ivert, 2018; Skogan & Maxfield, 1981), a notion coined “female fear” (Gordon & Riger, 1989; Silva & Wright, 2014). Research regarding fear of sexual victimization has typically focused on samples of young, college age, women who are found to fear victimization at a greater level than older women (Mellgren & Ivert, 2018). However, at present, there is an absence of literature examining whether older women with daughters have higher levels of fear relating to their daughter's risk of sexual violence.

Experiences of sexually aggressive behaviors, such as being followed, ogled, sexualized, and cat-called, are linked to increased fear (Szymanski & Bissonette, 2020; Holgate, 1989; Lane, 2012; Mellgren & Ivert, 2018). The unpredictable nature of interactions with male strangers explains this correlation, whereby harassment translates as a potential threat and leads to feeling unsafe in the public arena (Szymanski & Bissonette, 2020; Davidson et al., 2016). Feminist literature reports that this relationship between aggression and fear is yet another tool to control and oppress women and serves as a reminder of women's vulnerable position in society (Brownmiller, 1975; Lane, 2012; Mellgren & Ivert, 2018).

Research has established that women adopt avoidance and self-precautionary behaviors to protect themselves from sexual violence (Gordon & Riger, 1989; Riger & Gordon, 1981). Avoidance behaviors isolate individuals from danger by restricting actions, such as staying home (Valentine, 1989). Self-precautionary behaviors minimize potential risk in the presence of danger (Riger & Gordon, 1981). Women are more likely to engage in risk management strategies to reduce their risk of sexual violence when outside of the home environment, particularly at night (Ceccato & Loukaitou-Sideris, 2021; Stark & Meschik, 2018). Strategies include making more strategic outfit choices, changing routes, carrying forms of repellent, and staying home (Ceccato & Loukaitou-Sideris, 2021; Stafford & Pettersson, 2004; Stark & Meschik, 2018; Vera-Grey & Kelly, 2020).

### The Transposition of Fear From Mothers to Daughters

Parents play a critical role in the sexual socialization of their children (Edwards et al., 2022; Evans et al., 2019; Cederbaum & Hutchinson, 2016; Rossetto & Tollison, 2017) as they model sex and gender-appropriate behaviors, cultural stereotypes, values, and beliefs (Rossetto & Tollison, 2017). Exploring perceptions of sexual violence held by parents is critical as they are pivotal figures in facilitating their children receiving sexual violence education, attending prevention programs, and providing instrumental support (Edwards et al., 2022, Evans et al., 2019).

Scholars argue that fear of sexual violence is not a inherent aspect of adulthood but is often integrated through a child's upbringing via parental influence and socialization (Rogers, 2016). Parents may introduce fear of sexual violence by providing warnings related to their daughter's vulnerability in the public sphere (Gordon & Riger, 1989; Silva & Wright, 2014), particularly through rhetoric that sexual violence is inescapable (Rossetto & Tollison, 2017). Mothers more commonly introduce conversations regarding risk (Evans et al., 2019; Harris, 2016; Sneed et al., 2013), and daughters are significantly more likely to be advised of the risks and consequences of sexual behavior than sons (Aronowitz & Agbeshie, 2012; Flores & Barroso, 2017). Despite these findings, discussions of sexual violence are often considered taboo within households, and result in vague, non-descript conversations within the family (Rossetto & Tollison, 2017) that perpetuate harmful stereotypes and rape myths, or spread misinformation (Burt, 1980, 1998; Gordon & Riger, 1989; Silva & Wright, 2014).

## The Current Study

The current study qualitatively examined the experiences and perceptions of sexual violence risk across two samples of women between June–November 2022. Comparisons of interview responses from young women (*n *= 10) and mothers of young women (*n *= 10) explored how these groups experience and manage sexual violence risk. Specifically, this study explores risk-based fear, coping and management strategies, and the role of policy in protecting women. The current study has two research aims: (a) examine how UK-based young women experience and manage fear related to sexual violence and (b) understand how UK-based mothers of young women perceive and manage sexual violence risk to their daughter(s).

To further understand this topic, young women and mothers of young women who live in the UK participated in interviews conducted between June and November 2022. Notably, these young women were not the daughters of the interviewed mothers and should be understood as two separate samples. Interviews were transcribed and coded using reflexive thematic analysis that included both manifest and latent coding procedures. Previous studies relating to the mother–daughter dynamic, sexual violence risk, and policy implications contextualize the findings. The study followed ethical principles outlined in the Tri-Council Policy Statement 2, which governs research conducted at Canadian postsecondary institutions. Simon Fraser University's Office of Research Ethics designated the studies as minimal risk on June 7, 2022 (young women study) and September 1, 2022 (mothers study).

## Methods

### Sample

Participants were recruited using a purposive and snowball sampling framework. Inclusion criteria for both samples stipulated that participants must identify as female, be aged (or have a daughter aged) between 18 and 30 years old, and live in the UK. The sample of young women consisted of 10 women, all aged 25, situated in various locations across the UK,^1^ six of whom were in long-term relationships with men. Participants lived with their parents or with roommates. Participating mothers consisted of 10 women, aged between 50–62 years, living in various locations in the United Kingdom.^2^ All 10 mothers were married to men and lived with their partner and children or just their partner. Participants were asked to choose a pseudonym to be referred to, across the following paper (-M) signifies the participant was from the sample of mothers, and (-YW) from the young women.

### Recruitment and Interviews

Participants were recruited via an online flyer posted to the researcher's publicly accessible Twitter and Facebook, through email, or by snowball sampling from previously interviewed participants. Informed consent, including promises of confidentiality, anonymity, and voluntariness, was obtained prior to recording interviews. Semi-structured interviews followed an interview guide (see Appendices A and B). The interview guide for young women covered three central themes: sexual violence risk, risk management strategies, and confidence in sexual violence policy. Themes in the mothers’ guide included: sexual violence risk, daughter's risk management strategies, parental risk management strategies, and confidence in sexual violence policy. When possible, the interview guides across both samples were mirrored.

### Coding Procedure

**Figure 1.**
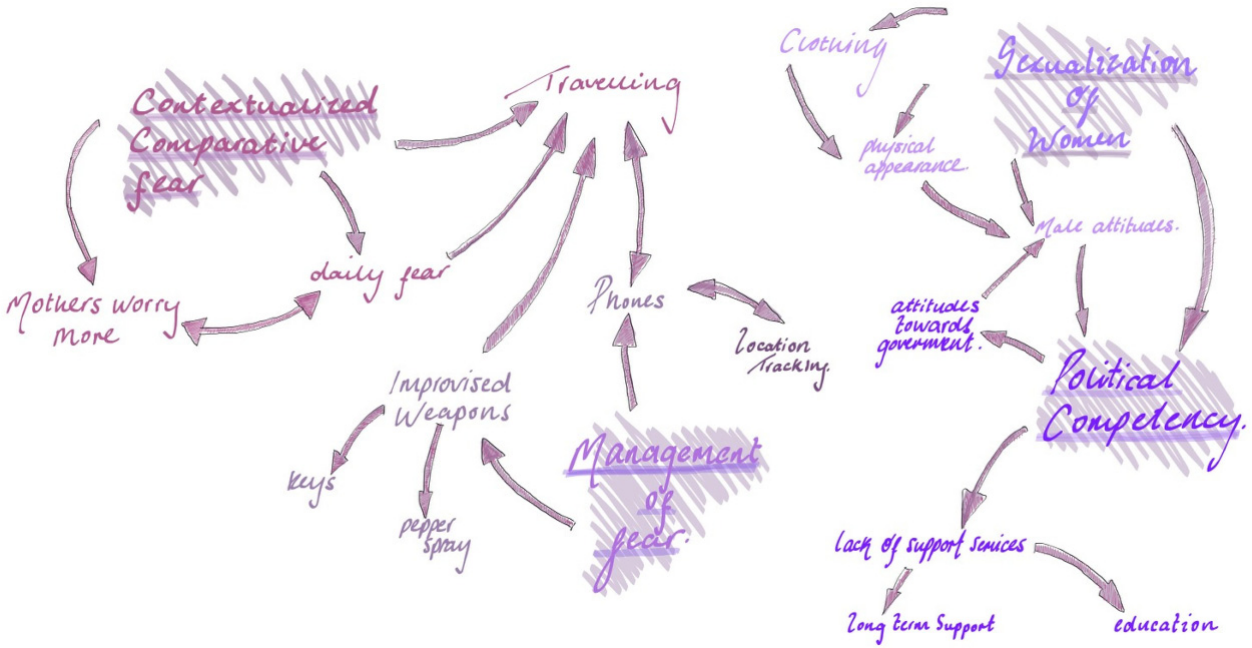
Thematic map.

Interviews were conducted on Zoom, audio recorded, transcribed, and subsequently verified by listening back to the audio and quality checking the accuracy. Transcripts were coded and analyzed using NVivo, a qualitative data management software. Latent and manifest coding was used to identify prominent themes. These combined processes produced a deeper exploration and interpretation of participants’ experiences and are viewed as a more valid and reliable coding framework (Cash & Snider, 2014; Goel & Pirolli, 1992). The generation of themes followed Braun and Clarke's (2012, 2020) reflexive thematic analysis approach, using the format of a thematic map shown in Figure 1.

Following preliminary coding procedures transcripts were reviewed to confirm that the final thematic development accurately reflected participants’ views.

Ultimately four themes were identified: (a) Comparative Fear, (b) Management of Fear, (c) The Sexualization of Women, and (d) Political Competency. Under these four themes, there were 11 subthemes which are shown in Table 1.

**Table 1.** Key Themes and Subthemes.

Key themes	1. Comparative fear	2. Management of fear	3. The sexualization of women	4. Political competency
Subthemes	1a. Mothers worry more versus young women's daily fear	2a. Phones as mechanisms of protection	3a. Physical appearance	4a. Attitudes toward the government
	1b. Concerns over travel	2b. Improvised weapons as mechanisms of protection	3b. Male attitudes and gendered norms	4b. Lack of support services

## Findings

Study findings first introduce comparative fear, which explores conditions that influence participants’ fear of sexual violence, followed by a discussion of how they manage that fear. The sexualization of women and the effects of this sexualization are considered, before concluding with a commentary of the political (in)competency of the UK government to respond to sexual violence guided by the participants.

### Comparative Fear

The following theme shows how mothers of young women and young women themselves consider their threat and fear of sexual violence. There were numerous discrepancies between these considerations; for example, mothers minimized the level of fear they believed young women to have about their risk of victimization. Despite these disparities, the two groups some shared experiences; for example, both took proactive steps to avoid victimization to themselves and/or their daughters.

#### Mothers Worry More vs. Young Women's Daily Fear

Mothers viewed their daughters’ behaviors as riskier and believed them to be less aware of sexual violence risk. Conversely, young women understood their risk to be pervasive, thought about it daily, and took alleviating precautions. Mothers frequently identified perceived “risky” behavior from their daughters: “The fact she would walk through this park where she knew a sexual assault had taken place was concerning to me” (Mischa-M). Often, the mothers' fear of their daughter's potential sexual violence victimization manifested as criticisms towards their behavior. Here, it was evident that fear exhibited by mothers was projected onto self-preservation tactics necessary for their daughters to adopt. Across the interviews, mothers believed they paid more attention to “high-risk behavior”: “I probably see Boogeyman around every corner, and I think she would see her risk as much less” (Theresa-M). They rooted these beliefs in maternal instincts: “She probably would never realize how worried we are as mothers until they become parents themselves” (Abigail-M). It is important to note here, that none of the daughters of the interviewed women had children. As such, the mothers validated their concerns existing at a greater level than their daughters’ and highlighted the belief that their daughters would only be capable of maternal consideration of risk if they had daughters of their own.

Mothers consistently exhibited fear through imagining a police officer coming to their door to report harm to their daughter, as highlighted by a recent memory shared by Gabby-M:

I knew they were going out and at 3:15AM the doorbell was ringing and there was a helicopter flood light on our front door. When I got up and looked out the window it looked like a police car and I honestly thought she was dead, I really did… It is your worst nightmare to get a knock at the door at 3:15AM.

The majority of interviews with mothers raised similar scenarios. Mischa-M stated “I didn’t want to be one of those mothers where the police come to the door.” This imagery highlights the pervasive panic that exists among mothers who have daughters that experience independence from the household—particularly in the nighttime economy—and the repercussions of this increased liberty. However, the mothers also admitted that their daughters did not welcome their heightened fear: “It does panic me a bit, but they think I worry too much” (Sasha-M). As such, receptivity of fear was a legitimate concern in which, mothers believed projection of fear would push their daughters further into risky situations. However, the notion that mothers worry too much about sexual violence was not evident across the findings with young women.

Despite the mothers' confidence that they feared risk to their daughters at a greater level, the young women described sexual violence anxiety as a constant in their lives: “I definitely feel like even when I am out in the day-time, I am still scared of everything” (Dolly-YW). Experience of sexual harassment was often cited as a key contributing factor to the young women's prevalence of fear, “I don’t know a single female that doesn’t have an experience where they have been harassed, touched inappropriately, cat-called down the street, followed home. I don’t know a single person that doesn’t have at least one of those stories to tell” (Cheryl-YW). Recounts of sexual harassment were prolific among the sample of young women, who identified that these experiences heightened their fear of sexual violence victimization. These findings indicated misunderstanding between the experiences of mothers and young women, indicating discrepancies in transparency of communication while highlighting the levels of fear across both groups.

#### Concerns Over Travel

Both groups felt traveling somewhere on their own, particularly in the dark, presented the greatest risk. The young women used avoidance behaviors after daylight and when they were alone: “After work I don’t do anything because it is dark, and I don’t want to worry about getting home in the dark” (Gemma-YW). Pervasively, these participants identified that darkness and limited methods of transport home increased their fear—and perceived likelihood—of being victimized. The young women also identified avoiding certain locations: “I would never walk through a park at night” (Lana-YW). These findings demonstrated that their experiences of, or information they were given about, sexual violence contributed to their identification of risky spaces. The mothers shared similar traveling concerns as Mischa-M acknowledged: “I think any mother would worry about her daughter walking home at night.”

Fear of isolated travel applied to mothers', their daughters', and the young women's safety. Jane-M contextualized her own experiences of sexual violence: “Stranger rape is very rare, but we fear it when we walk ourselves down a dark road. I have been attacked myself a few times quite unpleasantly. That is a real fear, but it is rare.” Despite recognizing that stranger-based violence is not commonplace, personal experiences of sexual violence may have led to heightened concern for their daughter's safety. To mitigate fear and risk, the mothers shared pre-emptive discussions of risk management strategies with their daughters: “I would just make sure she always knew that if they ever had a problem with getting a taxi, and even if I was asleep, she can ring me, and I will come and get her” (Samantha-M). They further acknowledged that when their daughters left the house at night “we were basically on call” (Mischa-M). Offering rides home served to protect their daughters from sexual violence, and eased the mothers’ concerns, as Theresa-M highlighted: “We provide taxiing services for her, so I think we provide an invisible cushion which really is our way of protecting her.” The mothers adopted strategies that both minimized their concerns and protected their daughters from violence. In these instances, the mothers emphasized how the pressure to prevent their daughters’ victimization was inescapable when they were uncertain about their ability to make it home safely.

In a similar vein, the young women consistently recognized they used travel-based risk management strategies to mitigate their fear and risk of sexual violence: “I definitely change my route loads of times, there has been points where I just get the bus because the stress in my head is not worth it” (Ethel-YW). As such, the young women highlighted precautions they put in place to reduce victimization, regardless of the time or effort needed. These tactics suggest that maternal early intervention may influence young women to engage in protective behaviors. However, these considerations do not account for the various other pathways in which young women learn and communicate about ways to mitigate sexual violence risk (e.g., the media, peers, school education). Ultimately, all participants viewed their hesitancy to go out on their own after dark as a point of frustration related to the social isolation of women: “I am so furious about the fact the nights are dark now therefore women's freedom is limited. I am infuriated by it. Clocks have gone back, back in your cage women in the UK” (Gabby-M). Both samples identified the level of fear, anger, and effort they experience when considering risk of sexual violence, emphasizing how arduous it is to be consistently conscious of victimization.

### Management of Fear

“Management of fear” outlines the various mechanisms participants used to protect themselves—or their daughters—from sexual violence and ease their fears. Namely, participants felt that ability to contact loved ones or track locations through mobile phone devices was a particularly useful mechanism. Participants also discussed the importance of being prepared for self-defense through the use of protective household items in response to active threat. These improvised weapons included items such as keys, pencils, or any other available sharp object.

#### Phones as Mechanisms of Protection

One common protection measure introduced by both the mothers and young women was the use of phones, specifically location-tracking services. Young women described a sense of relief from sharing their location with parents, partners, or friends: “I often put my location on WhatsApp which makes me feel a lot safer that someone knows where I am” (Louise-YW). Experiences such as this demonstrated the use of self-soothing mechanisms that helped women feel safer when leaving the home. Similarly, the mothers explained how their concerns were eased when they could view their daughter's location:

We have a Life App, if she is going on a date or going somewhere she will tell me … then I can see where she is on this app. Sometimes she has said if I am not home by such and such a time can you call me? Although I don’t abuse the app, if she needs me to be there or I have any concerns then I can be there. (Abigail-M)

Here Abigal-M identifies a consistent internalized pressure among mothers and young women to acknowledge risk and adopt mechanisms that could prevent victimization. Another mother, Jane, disclosed an incident when location-tracking services were pivotal in a sexually violent incident her daughter was involved in:

There was one occasion when she was sexually assaulted quite badly, she was rescued because she had Snap Maps and she couldn’t get her phone to send any messages, but she could grab it and stab it wildly. Some of her friends got these weird messages and they looked at Snap Maps and could see where she was, so they turned up. The police came afterwards.

As such, location-tracking services not only served to mitigate sexual violence-based fear, but also served as a legitimate mechanism for identification when victimization did occur.

#### Improvised Weapons as Mechanisms of Protection

Improvised weapons were also seen as physical mechanisms of protection across both samples. Young women discussed the use of pepper spray and other potentially protective tools. However, they primarily identified house keys as their main method of self-defense: “I put keys in my hand and between my fingers” (Ethel-YW). Even participants that did not disclose experience(s) of sexual violence expressed that when they were alone they were consistently evaluating what tools could be used in the event that an attack occurred.

Mothers also recognized the value of improvised weapons, “You could carry a weapon, a pencil in the eye would be quite effective” (Cathy-M). Whilst also recommending these protective mechanisms to their daughters, the mothers also used these tactics for their own safety: “I wouldn’t walk around the streets on my own without something sharp in my hand as preparation, which is ridiculous. But it has never stopped me” (Abigail-M). Across both samples, participants often reduced their use of protective mechanisms to being “ridiculous” despite the pervasive fear—and victimization—experienced. The level of fear experienced by both mothers and young women resulted in feelings of responsiblility to protect themselves in their daily lives; they recognized that they could not avoid all potentially violent situations and so must employ protective mechanisms to feel and stay safe.

### The Sexualization of Women

Participants believed that personal experience of sexualization—and women more widely—contributed to their levels of fear and risk. They discussed remaining conscious of their—or their daughters’—clothing when leaving the house and took precautions to alleviate these perceived risks, such as dressing less ‘provocatively'. However, the pressure to adopt such mechanisms was a point of frustration and sadness. Participants also noted that gender disparities across fear and victimization were formative to their attitudes towards female sexual violence. Specifically, they felt that patriarchal attitudes and misinformation amongst men cause misunderstanding—and even a proliferation—of the female experience of sexual violence fear, risk, and victimization.

#### Physical Appearance

One prominent subtheme was the association between young women's clothing and physical appearance with sexual violence risk. Many participants made this link and as a result, tailored outfits to reduce their victimization: “I definitely wear different clothing if I know I am walking somewhere by myself… If I was going to London, I would probably dress a bit more plain and I wouldn’t wear a shorter top” (Helen-YW). For some, this risk was locationally specific to large metropolitan cities such as London. Participants in these areas often felt more at risk of stranger violence due to the population size and distance from their hometowns.

Mothers saw clothing and risk as a point of conflict within their role as a parent. Mothers wanted independence for their daughters yet feared the risk associated with “provocative” clothing. Julia-M acknowledged this conflict:

The girls [her daughters] used to wear their skirts so short for school, but they should be able to do that if they want but unfortunately it is a concern, but why? You are pulled both ways, telling them they shouldn’t be wearing that because men might get the wrong idea… You don’t want to make them feel as if they shouldn’t dress how they want to dress just because there are vile men around. It is difficult getting that balance.

Balancing concerns with the personal freedom of their daughter's was prominent across the majority of interviews with the mothers, and few identified a solution to this internal conflict.

Discussions of outfits and the sexualization of women were often rooted in a wider discussion of a gendered society, as mentioned by Nancy-YW, “I know we have to grow a lot as a society in terms of feminism because no woman should be at risk of rape if she wears a short skirt, or she's pissed [intoxicated].” Participants were cognizant of gendered considerations of sexual violence risk and expressed particular contempt at this.

#### Male Attitudes and Gendered Norms

Both samples discussed experiences of harmful male behaviors and the need for increased education on sexual conduct and ajustment to patriarchal attitudes and misogynistic behaviors. The young women and mothers believed that men lack consideration for women's sexual violence risk and/or experiences. Samantha-M stated: “I don’t think men appreciate what it is like growing up as a woman.” This notion was shared by many of the young women, including Phoebe: “Men don’t realize how much we have to think about … they just don’t see the effect that can have when these little things just mount up and you feel so harassed all the time.” In this context, women alluded to feelings of isolation in their experiences of sexual violence fear and victimization and did not feel supported by the men in their social circles.

Disparity across genders regarding the adoption of risk management strategies was also acknowledged across both the mothers and young women, as Louise-YW emphasized: “I think it is just really sad and unfair that women have to do this when men don’t have to give it a second thought.” Alongside sadness, participants expressed anger and frustration at male-perpetrated violence and connected this to women's right to be safe:

It pisses me off deeply. I mean, who do men with their dicks think they are? It profoundly annoys me and leaves me almost speechless with annoyance and upset. Each of us are responsible for our needs so why do you think dominating and harming someone else is an effective coping strategy? Who the fuck do you think you are? So, gaining the streets back for women and all that kind of stuff, people have a right to be safe. (Gabby-M)

Alongside depictions of anger, participants described the need for women to exist with freedom, safety, and comfort in social spaces without fear of sexual violence.

Amongst participants, gendered norms and a lack of understanding of sexual violence were tethered to the need for better educational measures targeting the experiences of women: “Education in schools, young boys from a young age to know what women have to go through. Education and being more aware of it” (Gemma-YW). Mothers expressed similar sentiments: “There is a lot of push toward putting the responsibility on men and boys and educating them, everything has to start at school and the attitude of men has to change for women to feel safe” (Julia-M). Participants recognized that women are cognizant of the societal disadvantages they face when it comes to violence. However, they emphasized a disconnect in awareness of sexual violence risk across genders and a need for gender equity in these considerations.

### Political Competency

The following theme presents participants’ beliefs that the UK government has failed to protect women from sexual violence. Specifically, participants felt that overt sexist attitudes among members of parliament facilitated widespread misogyny and enhanced fears of victimization. Participants also spoke of their own experiences—or knowledge—of victimization and highlighted a lack of available support services as a key concern for tackling this issue.

#### Attitudes Toward the Government

Participants were critical and disheartened when asked about the government's ability to protect women from sexual violence. Parliament's gendered attitudes were evident in the treatment of (at the time of interview) deputy labor leader Angela Rayner (deputy prime minister as of July 2024). It is important to note here that the interviews took place in 2022 at which time the UK was goverened by the conservative party:

The attitude in the government towards women is not very helpful in cracking down on violence against women, for example Angela Rayner when the MPs were accusing her of trying to distract Boris Johnson by having her legs open. With those sorts of attitudes and perceptions of women and their sexuality, I don’t feel very confident in their ability to decrease sexual violence. (Phoebe-M)

Discussions of the sexualization of Angela Rayner were commonplace across the interviews:

Women's rights in the UK have gone back twenty years in the past five years, we’ve gone back forty years in the past fifteen years when they’ve [the conservative party] been in power. I have seen it all the time. The treatment of Angela Rayner as the deputy labour leader is absolutely appalling. If you want to see misogyny written large in our society in the UK and what they really think of women look at how they treat her. (Gabby-M)

Participants exemplified oppresive gendered attitudes that perpetuate sexually violent behaviors, regardless of social and political positioning, that exist within the UK through their attention to the treatment of Angela Rayner. Participants also felt the demographic and social characteristics of the conservative government were key factors facilitating the continued discrimination and risk of sexual violence experienced by UK women. Theresa-M explained: “I have to think about who our government is. Our government is mainly public school overprivileged boys and they don’t like any kind of vulnerability. They are very entitled, and they are very entitled to women's bodies.” Participants emphasized the belief that governmental actions and attitudes towards women influences societal gendered perspectives. As such, they expressed little hope in a reduction in sexual violence prevalence without significant changes at the higher level. These discussions highlight the lack of confidence that both samples held toward the conservative government's ability to appropriately address sexual violence amongst women.

#### Lack of Support Services

The most prominent criticism of the conservative government was the absence of accessible and available sexual violence support services. Violet-YM reflected on this lack of support: “In terms of the current [conservative] government, I can’t think of any mention of practical things [they do] to keep women more safe.” As such, these women often felt they had no pathways to seek help for their own—or others’—experiences of sexual violence at the macro-level. Another participant, employed in a sexual violence-related field, highlighed the lack of long-term support for victims and the need for improved systems to respond to these issues:

When you deal with [sexual] assault you need to see it as a long-term issue that needs to be dealt by experienced staff members. You get help that splits into “do five sessions and change your thoughts”, you need well informed ongoing work about how you trust again, how you deal with trauma, how you deal with PTSD, how you deal with all those consequences about letting people touch you again. That can’t be done in a few sessions. (Theresa-M)

This participant spoke to their own experience of referring victims of sexual violence to support services and expressed disappointment in the pre-existing systems that appeared to be short-term responses to long-lasting trauma. Both samples had a diverse education and employment background, yet all participants viewed the conservative government as incompetent in protecting women. Another mother, whose daughter had experienced sexual violence, shared their own experience of governmental ineptitude:

It is not very good, there is not enough support. When she [her daughter] went through it, the only people we had were the police and I paid for a private counsellor for her to try and make sense of what happened and why. There didn’t seem to be anything else I could tap into. (Maggie-M)

These first-hand experiences of unavailable sexual violence support services highlight the absence of a clear strategy to address these prolific concerns in the UK.

Some participants expressed a desire to seek victim services but faced barriers to access and/or were unaware of available support. Other participants, who did not disclose experiences of sexual violence or the need for services, imagined that services would be inaccessible/unavailable.

## Discussion

### Prevalence, Protection, and Misunderstandings of Sexual Violence Fear

Across both samples, findings showed that women fear sexual violence in a pervasive way that interrupts their ability to complete basic tasks—such as walking home—without being conscious of their risk of sexual violence. These fears reflect “availability heuristic,” in which the likelihood of an event occurring is related to the ease at which it can be recalled (Tversky & Kahneman, 1974). Media overrepresentations of sexual violence, particularly violence perpetrated by strangers (Peelo et al., 2004; Waters et al., 2017) feed directly into women's levels of fear as they can more readily recall media coverage of sexually dangerous situations (Chermak, 1995; Gravel et al., 2017; Waters et al., 2017; Wong & Lee, 2021). However, literature has established that women routinely consider their risk to be lower when comparing themselves to others in their demographic (Chapin & Pierce, 2012; Messman-Moore & Salim, 2019). Expressions of fear among the sample of mothers and young women contradict this literature related to optimistic bias, which asserts a divergence between perceived personal risk and risk to others (Vitek et al., 2018). The sample of young women described extensive fear of sexual violence victimization and the mothers believed their daughters were at the same risk as other young women. Equal distribution of risk is a promising finding, as optimistic bias can lead to a harmful reluctance to engage in self-protective risk responses (Messman-Moore & Salim, 2019).

Outside of media consumption, there are myriad ways in which women may be made more conscious of their sexual violence risk—such as dialogue among peers. As demonstrated in the findings, the young women discussed sexual violence risk among their friends. These conversations may feed directly into perceptions of one's own risk—particularly if recounting experiences with someone from a similar demographic. Here, it is important to reflect on the role of homophily and consider how people are often confined in their learning, beliefs, and interactions due to predominantly associating with people who share similar characteristics (Hershcovis et al., 2021). As such, fear of sexual violence and the need to adopt risk management strategies may be exacerbated among young women—and mothers of young women—due to shared beliefs and experiences.

The participants indicated pervasive fear of sexual violence. However, mothers in this study often mitigated the level of fear young women experience and the risk managment strategies they adopt. Young women routinely cited daily and pervasive fear yet the sample of mothers did not believe their daughters appropriately implemented strategies to lower the likelihood of victimization. These findings highlighted a potential disconnect among mothers of young women and young women themselves in which fear of sexual violence—and response to this fear—may not be adequately discussed in familial settings. The mothers’ belief that young women do not consider their risks may reflect the “taboo” nature of discussing sexual violence within the family (Rossetto & Tollison, 2017) and result in a misunderstanding of fear levels experienced and risk management strategies adopted across mothers and daughters. This confusion emphasizes the need for better, and more transparent, communication channels between families targeted towards sexual violence discussion. Furthermore, the availablility heuristic recognizes that parental exposure to sexual violence information increases fear for their children (Mancini et al., 2010; Zgoba, 2004) and may explain why mothers believe they fear risk to their daughters at a greater level. Altruistic fear may further explain maternal concern (Warr, 1992) whereby individuals fear the victimization of another person at an increased rate, in this case, their daughter. Altruistic fear is prominent within the family dynamic and has been found to be more profound, intense, and frequent than personal fear of victimization (Drakulich, 2014; Lee et al., 2022; Vozmediano et al., 2017; Warr & Ellison, 2000). The dichotomy between mothers who believe they consider risk to their daughters more carefully than their daughters themselves do and the young women who discussed how prevalent their fear and risk was unanticipated and therefore, of great interest. To date, no studies that have examined the perceptions of sexual violence victimization for women and their daughters. As such, these findings contribute to the understanding of sexual violence perceptions across the mother–daughter dyad and demonstrate the need to explore this topic further.

Fear of sexual violence caused participants to engage in multiple protective behaviors, including using location-tracking services and improvised weapons. Participants' experiences of misogyny and lack of government intervention compelled them to absorb the responsibility of victimization prevention. Although research supporting the utility of location-tracking services in reducing victimization is limited, the use of protective personal safety devices has been increasing (Maxwell et al., 2019). Women are more likely to download personal safety apps that include tracking services and feel safer when using such apps (McCarthy et al., 2016). Specifically, app creators have been commended for facilitating increased feelings of empowerment and safety amongst women (Maxwell et al., 2019). Despite cited benefits, numerous critiques of these apps exist, e.g., they are reactive rather than proactive, they may reduce the practice of other protective mechanisms, and they may increase the potential for family members or intimate partners to abuse these services (Hasinoff, 2016; Maxwell et al., 2019; McCarthy et al., 2016).

Participants identified the use of sharper household objects as precautionary weapons. The UK has criminalized many other preventative tools, for example, the possession of pepper spray is considered a criminal offense under the *Offensive Weapons Act* (2019). The only legal self-defense mechanism allowed in the UK is a rape alarm (West Yorkshire Police, 2024), which limits women's ability to physically protect themselves from violence. Participants may have felt the responsibility of producing their own methods of protection by carrying other sharp objects, such as keys or pencils that can be used in moments of crisis in response to this legislation.

### Sexualization and Clothing

Physical appearance contributed to risk perception among the sample; for example, the young women recognized they often modify their clothing to reduce risk of victimization. The sample of mothers also expressed concern when their daughter's wore clothes considered to be more revealing. These perspectives align with studies that identify risk factors for sexual violence including “sexiness,” and question victim credibility when their clothing is considered provocative (Frazier, 2020; Moor, 2010). Given this rhetoric, participants’ association of clothing with increased risk that could be mitigated by altering their outfits was expected. These victim-centric attitudes also help explain the contention mothers felt about their daughter's clothing choices. Mothers wanted their daughters to have autonomy and freedom to dress themselves as they choose but feared the association between attire and sexual violence risk. This finding reflects victim-blaming narratives that argue provocative clothing is a high-risk behavior (Fileborn, 2016; Frazier, 2020; Moor, 2010). Given the role of parents in informing and developing their children's sexual and gendered attitudes, it is important to minimize the association of sexual victimization with physical appearance. This association facilitates the harmful rhetoric that appearance invites victimization, a narrative that has been found across familial discussions on sexual violence (Rossetto & Tollison, 2017). Reducing weight on this association could potentially reduce self-blaming attitudes. As observed in the current study, risk-mitigating behaviors continue to be an important protective factor for women. However, it is important to balance discussions surrounding perpetration and victimization, ensuring that victimization is not considered to be inevitable, invited, and the responsibility of the individual (Frazier, 2020). Going forward, familial sexual communication should contain fluid dialogues relating to gender, sexuality, and feminism that positively influence young people's perceptions of their self, body, and identity that aims to reduce tolerance and perpetration of sexually aggressive behaviors (Rossetto & Tollison, 2017).

Importantly, rhetoric linking sexual violence risk with phyiscal appearance exists outside of familial discussions. Specifically, news media has been found to perpetuate numerous rape myths including the relationship between clothing and sexual violence victimization (Merken & James, 2020; O’Hara, 2012). As such, it is not solely the familial environment that is formative of women's perceptions of sexual violence risk, but rather a combination of all pathways in which women learn and communicate about sexualization and risk of sexual violence.

### The Role of the Government

When reflecting on the governance of the UK at the time of the interviews, participants described witnessing sexually inappropriate behavior from politicians and their ability to influence perceptions of sexual and gendered norms. In this context participants gravitated towards the treatment of former labor deputy party leader (current deputy prime minister) Angela Rayner. Studies examining the gendered climate of the UK government found women in politics are frequently stereotyped, ignored, and sexualized (Harmer, 2021; Krook, 2018; Krook & Restrepo Sanin, 2019; Mantilla, 2015). Modeling misogyny and sexism contribute to sexually aggressive behaviors, and scholars have long argued that the existence of these attitudes perpetuate extreme typologies of sexual violence (Anitha & Lewis, 2018; Bates, 2016). As such, gendered discrimination at the government level may create a trickle-down effect that endorses tolerance of harmful behaviors.

Participants also critiqued the lack of government endorsement of sexual violence support across the UK. Specifically, they felt the government was unresponsive to the concerns—and the actual victimization—of women. These feelings came from both participants’ perceptions of government response, but also lived experience of sexual violence victimization and the inaccessibility of support services following the event. The perceived lack of support may explain the increased use, or encouragement, of precautionary measures such as location-tracking apps and improvised weapons across the samples while highlighting a lack of policy-based intervention for structural factors that lead to sexual violence (Astrup, 2022; De Benedictis et al., 2019; Rainbow, 2021; Sundaram, 2018). Findings showed that mothers also absorb the duty of protection by tracking their daughter's location and offering rides home. These experiences highlight the protective burden women bear, whether at the personal level or by proxy, and the need for better structural protective and preventative measures introduced by the government. To reduce the burden placed on women for protecting themselves—and their family members—from sexual violence, greater educational policies should be implemented. Scholars have emphasized the importance of minimizing day-to-day misogynistic behaviors and street-level harassment through school-based education programs (Anitha & Lewis, 2018; Bates, 2016). Further research has demonstrated that school curriculums that incorporate education on gender-based violence and encourage active participation from students and the community can increase awareness of sexual violence, reduce violent behavior, and provide greater support for victims (Villardón-Gallego et al., 2023). As such, the UK government should consider increasing funding streams directed toward schools to enable the implementation of such programs.

### Strengths, Limitations, and Future Work

The current study contributes to an understudied field by examining the perceptions of sexual violence risk among mothers and young women within the UK. Despite bridging gaps in understanding, this study is not without limitations. Persons of other genders also experience sexual violence fear and victimization and their voices have not been included here. Future work might specifically consider the communication—and experience—of risk for transgender and gender non-conforming individuals within the family context. Additionally, the sample of young women identified as straight and were in relationships with the opposite sex. It would be beneficial to consider how the experiences of the current sample may be compared to women who are in same-sex relationships and how this population may have distinct sexual violence risk considerations not applicable to women in opposite-sex relationships. Furthermore, this study expands on previous research by focusing on the influence of mothers in sexual violence socialization, but it excludes the narrative and role of fathers within the family. Future studies should explore the perceptions, experience, and influence of father figures. Consistent with previous literature, participants centered discussions of sexual violence around that perpetrated by strangers as could be explained by media attention to this form of victimization. Another explanation for the focus on stranger violence may be rooted in participants’ potential discomfort with disclosing or discussing sexual violence perpetrated by individuals known to them. The context in which participants discussed sexual violence should be expanded to specifically include violence from acquaintances, former, and/or current intimate partners. Finally, this study did not interview mothers and their own daughters; as such the mothers’ contributions cannot be transferred directly to the participating young women. This study does, however, provide new insights into the similarities and differences across young women's and mothers-of-daughters’ perceptions, fear, and management of sexual violence.

## Conclusion

This study introduces new considerations for sexual violence-based literature and explores the transference of fear and risk management through the lens of mothers and young women. The findings demonstrate that the fear of sexual violence transcends generations of women in the United Kingdom and show that both mothers and young women identify this fear as pervasive and inhibiting. Findings also point to increased need for more transparent communication between mothers and daughters regarding attitudes and experiences of sexual violence. The participants identified consistent adoption of precautionary and avoidance-based management strategies to reduce their (or their daughter's) risk of sexual violence such as location-tracking services, improvised weapons, offering car rides home, and dressing modestly. Participants expressed anger and frustration at the gendered nature of society and voiced contempt at the conservative government's attitude and inaction towards women and sexual violence policy. Suggestions for the future of sexual violence policy include better gender-based education, increased availability of long-term support, and more concerted efforts into reducing day-to-day misogyny.

## Notes

1. Young women's locations included the following counties: Tyne & Wear, West Yorkshire, Essex, Hampshire, Oxfordshire, Greater London, and Nottinghamshire.

2. Mother's locations included: Tyne & Wear, Somerset, Devon, Greater London, Bristol, and South Yorkshire.
